# Long-term impact of a quality improvement program on unplanned extubation and clinical outcomes in adult intensive care units: a 24-year single-center observational study

**DOI:** 10.3389/fmed.2026.1772510

**Published:** 2026-06-08

**Authors:** I-Lin Tsai, Chun-Wei Kuo, Ching-Min Wang, Chen-Wei Wu, Chin-Ming Chen

**Affiliations:** 1 Department of Internal Medicine, Chi Mei Hospital, Liouying, Tainan, Taiwan; 2 Pediatric Department, Chi Mei Hospital, Liouying, Tainan, Taiwan; 3 Department of Intensive Care Medicine, Chi-Mei Medical Center, Tainan, Taiwan; 4 Department of Intensive Care Medicine, Chi Mei Hospital, Liouying, Tainan, Taiwan; 5 Center for Quality Management, Chi Mei Hospital, Liouying, Tainan, Taiwan

**Keywords:** continuous quality improvement, intensive care unit, mechanical ventilation, mortality, reintubation, unplanned extubation

## Abstract

**Introduction:**

Unplanned extubation (UE) remains an important patient safety issue in intensive care units (ICUs) and is associated with morbidity, prolonged mechanical ventilation, and mortality, particularly when reintubation is required. Long-term evidence on sustained quality improvement (QI) programs in adult ICUs remains limited. This study evaluated the association between a continuous QI program and UE incidence and identified factors associated with reintubation and mortality after UE.

**Methods:**

We conducted a single-center observational study at a tertiary medical center. UE events were prospectively registered from January 1, 2001, to December 31, 2024, and clinical data were retrospectively analyzed. The QI program implemented from 2001 onward included standardized procedures, sedation and weaning protocol revision, adjustment of physical restraint practices, high-risk patient management, team-based QI frameworks, improved endotracheal tube fixation, and an artificial intelligence–based weaning prediction system. UE incidence was assessed among mechanically ventilated patients. First-episode UE patients from 2009 to 2024 were analyzed to compare outcomes between patients with and without reintubation within 48 h. Multivariable logistic regression was used to identify factors associated with reintubation and in-hospital mortality.

**Results:**

Over 24 years, 1,574 UE episodes occurred among 64,346 ventilated patients, with an overall rate of 2.51 per 100 ventilated patients. The rate declined from 6.82 in 2001 to 0.65 per 100 ventilated patients in 2024 (*p* < 0.0001). Among 442 first-episode UE patients, 52.5% required reintubation within 48 h. Reintubation was associated with longer ICU and hospital stays and increased mortality. Factors associated with reintubation included higher Acute Physiology and Chronic Health Evaluation (APACHE) II scores, elevated respiratory rate and positive end-expiratory pressure, longer intubation duration, and not undergoing ventilator weaning at the time of UE. Mortality was associated with higher APACHE II scores, liver cirrhosis, malignancy, elevated fraction of inspired oxygen and minute ventilation, higher blood urea nitrogen levels, prolonged ICU stay, and reintubation.

**Conclusion:**

A sustained, bundled, multidisciplinary QI program was associated with a durable decline in UE over 24 years. Early risk stratification and targeted post-UE monitoring may help identify high-risk patients and guide post-event management in adult ICUs.

## Introduction

Acute respiratory failure is one of the most common organ dysfunctions among critically ill patients (1, 2), and more than half of them require endotracheal intubation with invasive mechanical ventilation (MV) for respiratory support in the intensive care unit (ICU) (3). Once the acute condition stabilizes, timely liberation from MV is essential to avoid complications associated with prolonged ventilation, such as ventilator-induced lung injury, ventilator-associated pneumonia, increased ICU or hospital stay, and higher health-care costs (4, 5). Although extubation is usually scheduled after a structured weaning process, unplanned extubation (UE)—defined as accidental or patient-induced removal of the endotracheal tube—remains a significant clinical challenge. Reported UE rates range from 3 to 23% across ICUs (6–12), and a failed UE requiring reintubation is associated with serious adverse events including aspiration pneumonia, bronchospasm, arrhythmia, respiratory failure, and even cardiac arrest (13–15).

Despite the implementation of various preventive measures, UE continues to occur and can substantially worsen clinical outcomes. Previous studies have identified multiple risk factors for UE, including patient agitation, altered consciousness, insufficient sedation protocols, oral intubation, tube-securing methods, and issues related to physical restraints (16–19). Recent evidence from a pediatric ICU cohort similarly identified agitation, night shift, intermittent sedation, restraint use, and higher nurse-to-patient ratio as determinants of UE, further supporting the role of modifiable care processes in UE prevention (20). These findings underscore that no single intervention is sufficient; instead, sustained, multidisciplinary quality improvement (QI) efforts are necessary to meaningfully reduce UE incidence. Comprehensive QI programs targeting modifiable processes—such as standardized tube fixation procedures, communication improvement, and enhanced surveillance—have been shown to decrease UE rates and improve safety in the ICU (12, 21, 22).

In addition to prevention, early recognition of high-risk patients after UE is essential (10, 11), as those requiring reintubation experience prolonged MV duration, longer ICU and hospital stays, and higher mortality (15, 23). Identifying prognostic factors associated with UE-related extubation failure and mortality can help clinicians optimize post-UE management and allocate resources appropriately. Therefore, in this study, we evaluated the long-term impact of a sustained QI program on reducing UE in the ICUs of a tertiary medical center from January 1, 2001, to December 31, 2024. In addition, using a robust longitudinal dataset from January 1, 2009, to December 31, 2024, we examined outcomes of successful versus failed UE, identified risk factors for reintubation within 48 h, and evaluated prognostic factors for mortality among patients experiencing UE.

## Methods

### Study design and patient selection

This study was conducted at Chi Mei Medical Center, a tertiary facility comprising 1,288 total beds and a 96-bed ICU, including 48 medical, 9 cardiac, and 39 surgical ICU beds. The study cohort included all patients experiencing a UE event. Data were prospectively registered and retrospectively analyzed from electronic medical records (EMR) extracted from a central databank from January 1, 2001 to December 31, 2024. To ensure data independence, analysis was restricted to the first UE episode per hospital admission. The study was approved by the Institutional Review Board (IRB) of Chi Mei Medical Center (IRB: 11410-L04). Given the retrospective nature of data collection from routine records, the requirement for informed consent was waived by the IRB of Chi Mei Medical Center.

The ICU operates with a highly structured, multidisciplinary coverage model, which includes intensivists, senior residents, nurses, respiratory therapists, dietitians, physical therapists, and clinical pharmacists. Operational consistency was maintained by sustaining a patient-to-nursing staff ratio of 2:1 across all scheduled shifts. The clinical load ensured each respiratory therapist cared for fewer than 10 mechanically ventilated patients per shift. The ICU team performed rounds at least once daily, and respiratory therapists were accountable for the execution of all MV weaning processes and spontaneous breathing trials (SBTs). The final decision for extubation was the exclusive responsibility of an attending intensivist. Successful extubation was defined as the absence of a requirement for reintubation within the first 48 h following the procedure.

### Improvement interventions

A continuous QI program aimed at reducing UE has been implemented since 2001 (Supplementary Figure S1). This program focused on nine key areas: standardization of procedures and communication training, revision of sedation and weaning protocols, modification of restraint strategy, establishment of a task force for high-risk patient identification and management, implementation of the Breakthrough Series (BTS) quality improvement model and Team Resource Management (TRM), adoption of an accountability strategy, use of novel endotracheal tube fixation methods, and artificial intelligence (AI) prediction for ventilator weaning (12, 24).

**Figure 1:**
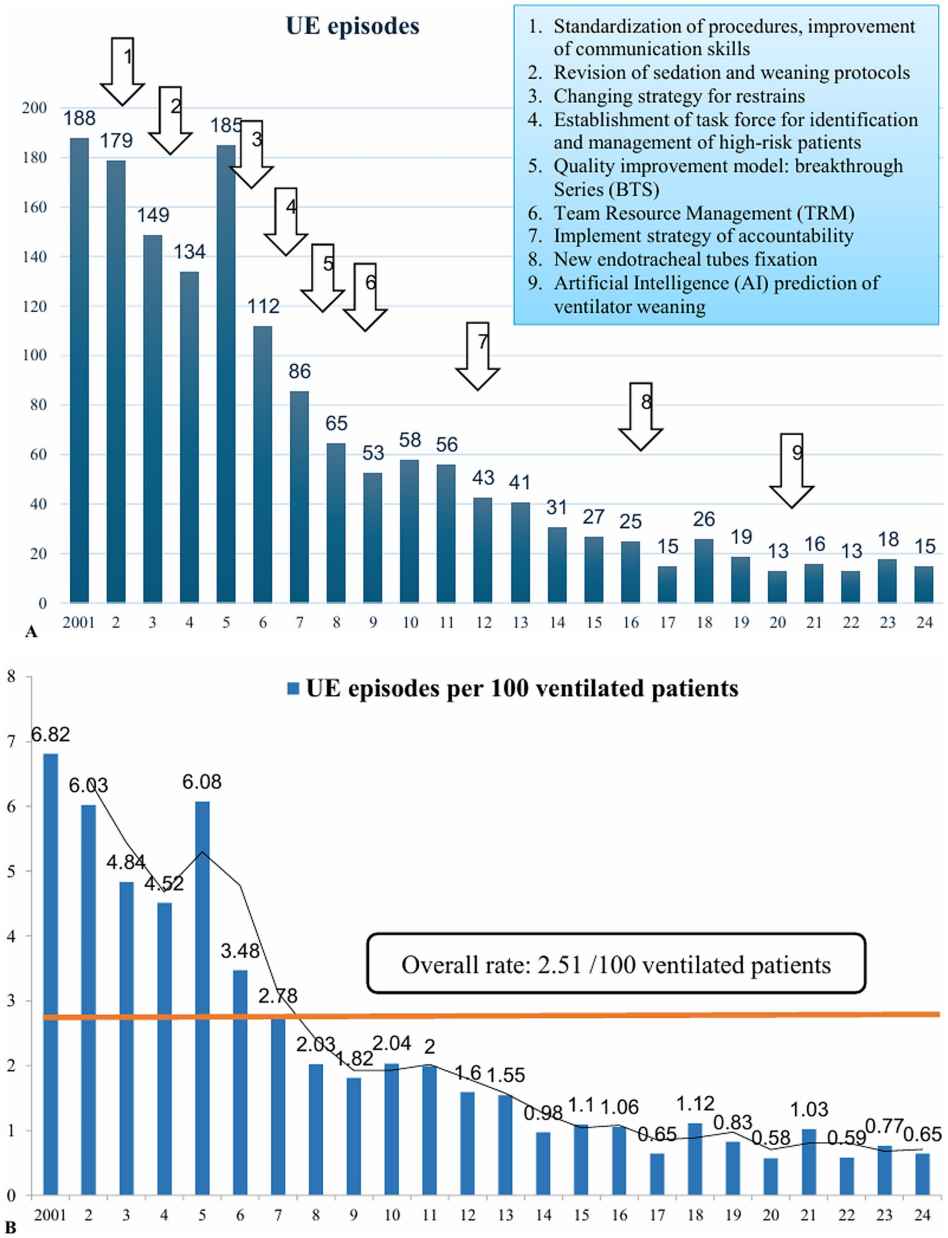
Annual trends in unplanned extubation (UE) and corresponding quality improvement interventions from 2001 to 2024. **(A)** Annual number of UE episodes. **(B)** Annual UE rate, expressed as UE episodes per 100 mechanically ventilated patients. Numbered arrows 1–9 indicate the timing of major continuous quality improvement interventions implemented during the study period.

The initial implementation year for each intervention and the methods used to execute the quality improvement program are detailed (Figure 1A: Interventions 1–9). In February 2002, initial standardization of procedures (Intervention 1) included educational programs for endotracheal tube (ETT) fixation, restraint strategies, and communication skills (e.g., using simple cartoon cards). In July 2005, a root cause analysis (RCA) (Intervention 3) attributed the unexpected increase in the UE rate to the recruitment of new nurses for ICU expansion and a subsequent change in patient restraint strategy, coinciding with a sharp rise in the restraint ratio from 1.58 to 20.30%. In January 2006, we established a high-risk patient task force, supported by computerized event recording and review processes (Intervention 4). Between 2008 and 2010, structural improvements continued with the introduction of new quality frameworks, including the BTS (Intervention 5) and TRM (Intervention 6). Since 2012, a non-punitive accountability strategy has been employed to drive recurrence prevention, highlighted by a public days-without-UE board (Intervention 7). In January 2014, physical fixation was enhanced by adopting the AnchorFast Guard tube fastener for ETT security (Intervention 8), replacing traditional tapes. Finally, in January 2020, the latest intervention involved introducing the AI prediction system for ventilator weaning (Intervention 9). This system used 7,019 electronic medical records from 2016 to 2019, processed via ETLV (Extract, Transform, Load, Validate). After testing various machine learning methods (random forest, logistic regression, and other machine learning methods) (24), the best model was established to predict successful weaning trials (25 features) and successful extubation (24 features). The system provides predictive estimates (e.g., a 69.65% successful weaning rate) based on real-time parameters such as fraction of inspired oxygen (FiO₂), positive end-expiratory pressure (PEEP), and peripheral oxygen saturation (SpO₂) to support clinical decision-making. All improvements were guided by the Plan–Do–Check–Act (PDCA) cycle.

### Data analysis and measurements

UE was defined as the unscheduled dislodgement or removal of the ETT in patients receiving MV. UE incidence was quantified as UE episodes per 100 mechanically ventilated patients. The failed UE rate was calculated as the proportion of UE patients requiring reintubation within 48 h.

Data were collected across four principal domains: (1) demographic and clinical characteristics, including age, sex, and ICU type; (2) disease severity scores, including the Acute Physiology and Chronic Health Evaluation (APACHE) II score, Therapeutic Intervention Scoring System (TISS) score, and Glasgow Coma Scale (GCS); (3) pre-extubation parameters, including vital signs, respiratory parameters, and laboratory examination results, such as respiratory rate, PEEP, and minute ventilation; and (4) clinical outcomes following UE, including ICU and hospital length of stay, hospital costs, reintubation within 48 h, and in-hospital mortality.

For mortality analysis, variables were considered according to their timing relative to the UE event. Demographic characteristics, disease severity scores, comorbidities, laboratory data, and respiratory parameters were collected before or at the time of UE. In contrast, reintubation within 48 h, total ICU length of stay, total hospital length of stay, and hospital costs were considered post-UE or outcome-related variables. These variables were evaluated as prognostic markers associated with in-hospital mortality and were not interpreted as causal predictors.

### Statistical analysis

Categorical variables are expressed as numbers and percentages. Continuous variables are expressed as means with standard deviations or medians with interquartile ranges, as appropriate. The linear trend test was used to assess the annual trend in the UE rate. For comparisons between non-survivors and survivors, continuous variables were analyzed using the independent-samples t-test for normally distributed data or the Mann–Whitney U test for non-normally distributed data. Categorical variables were compared using the chi-square test. Variables with statistical significance in univariate analyses (*p* < 0.05) were entered into multivariable logistic regression models to identify factors associated with failed UE and in-hospital mortality. In the mortality model, post-UE or outcome-related variables were interpreted as prognostic markers rather than causal predictors. All statistical analyses were performed using SPSS 26.0 for Windows (SPSS, Inc., Chicago, IL, United States). Statistical significance was set at a two-tailed *p* value of <0.05.

## Results

Over the 24-year study period, a total of 1,574 UE episodes occurred across 64,346 MV episodes. The overall UE rate was calculated at 2.51/100 ventilated patients (Figure 1B). The annual UE rate demonstrated a significant decline during the study period after implementation of the continuous QI program. Specifically, in 2001, there were 188 UE episodes, corresponding to a rate of 6.82 per 100 ventilated patients. By 2024, the annual number of episodes had decreased to 15, with the rate falling to 0.65 per 100 ventilated patients. A notable exception was observed in 2005, where the annual UE episodes and rate temporarily increased to 185 episodes and 6.08 per 100 ventilated patients, before resuming the downward trend. This temporary increase was attributed to the transfer of experienced critical care nurses to support the newly established branch hospital, resulting in a transient reduction in the quality of care within the main hospital’s ICU. Overall, the long-term trend analysis demonstrated a statistically significant change in the UE rate over the study period (*p* < 0.0001).

### Participants

From January 1, 2009, to December 31, 2024, 442 first-episode UE patients were included in the analysis; key characteristics are summarized in Table 1, and complete data are provided in Supplementary Table 1. The median age was 65.4 years, and 31.9% (n = 141) were female. Median disease severity scores were: APACHE II, 18; TISS, 28; and GCS, 10. The most common comorbidities were stroke (28.5%), coronary artery disease (24.4%), and diabetes (24.4%). UEs were more frequent during the day shift (35.3%) and evening shift (36.9%). Prior to UE, 35.1% (*n* = 155) exhibited agitation (Richmond Agitation-Sedation Scale [RASS] ≥ 2), and 65.2% (*n* = 288) received sedatives. Nearly half of UEs (49.1%) occurred during the weaning process (pressure support ≦14 cmH2O); the median intubation duration was 140.8 h. Patients who failed UE had higher APACHE II and TISS scores, lower GCS, a greater proportion of medical admissions, elevated respiratory rates, higher FiO₂, higher minute ventilation and PEEP, lower likelihood of undergoing ventilator weaning at the time of UE, and longer intubation duration prior to UE. When stratifying all UE patients by survival status, a similar pattern of baseline characteristics was observed in non-survivors, who also exhibited greater illness severity, worse physiologic profiles, and a higher proportion of patients not undergoing ventilator weaning at the time of UE.

**Table 1:** Key demographic, clinical, and pre-extubation characteristics of patients with unplanned extubation.

Variables	Total patients (*n* = 442)	Successful (*n* = 210)	Failed (*n* = 232)	*p*-value	Survival (*n* = 348)	Non-survival (*n* = 94)	*p*-value
Age (years)	65.4 ± 15.4	65.1 ± 1 5.3	65.7 ± 15.4	0.727	65.1 ± 16.0	66.7 ± 12.8	0.292
Female patients	141(31.9%)	66 (31.4)	75 (32.3)	0.840	115 (33.0)	26 (27.7)	0.320
Body mass index	23.8 ± 4.6	23.8 ± 4.6	23.9 ± 4.6	0.703	24.0 ± 4.6	23.3 ± 4.5	0.188
APACHE II score	18.2 ± 8.9	15.3 ± 7.9	20.7 ± 9.0	<0.001	16.7 ± 8.0.4	23.4 ± 8.7	<0.001
TISS Scales	28.0 ± 9.1	26.3 ± 8.2	29.4 ± 9.5	<0.001	27.0 ± 8.7	31.3 ± 9.6	<0.001
Glasgow Coma Scales	9.7 ± 4	10 ± 4	9 ± 4	<0.001	10 ± 4	10 ± 4	0.358
Medical patients	242 (54.8)	95 (45.2)	147 (63.4)	<0.001	177 (50.9)	65 (69.1)	0.002
Coronary artery disease	108 (24.4)	57 (27.1)	51 (22.0)	0.207	84 (24.1)	24 (22.2)	0.780
COPD	57 (12.9)	25 (11.9)	32 (13.8)	0.554	45 (12.9)	12 (12.8)	0.966
ESRD	40 (9.0)	15 (7.1)	25 (10.8)	0.184	18 (5.2)	22 (23.4)	<0.001
Liver cirrhosis	21 (4.8)	6 (2.9)	15 (6.5)	0.075	9 (2.6)	12 (12.8)	<0.001
Stroke	126 (28.5)	63 (30.0)	63 (27.2)	0.508	108 (31.0)	18 (19.1)	0.024
Cancer	50 (11.3)	20 (9.5)	30 (12.9)	0.259	25 (7.2)	25 (26.6)	<0.001
Mean arterial pressure (mmHg)	96.5 ± 17.9	97.4 ± 16.1	95.6 ± 19.6	0.352	98.1 ± 16.6	89.7 ± 21.8	0.004
Respiratory rate (breaths/min)	17.0 ± 5.6	16.3 ± 5.5	17.6 ± 5.7	0.015	16.7 ± 5.6	18.0 ± 5.7	0.039
FiO_2_ (%)	31 ± 13	29 ± 7	34 ± 17	<0.001	30 ± 9	38 ± 21	<0.001
PaO_2_/FiO_2_ (mmHg)	363.3 ± 180.2	374.9 ± 147.2	352.7 ± 205.2	0.206	374.3 ± 173.7	321.7 ± 198.4	0.014
Minute ventilation (L/min)	8.5 ± 3.1	8.0 ± 2.5	8.9 ± 3.5	0.002	8.2 ± 2.9	9.5 ± 3.4	0.001
PEEP (cmH_2_O)	5.9 ± 1.8	5.5 ± 1.2	6.4 ± 2.0	<0.001	5.7 ± 1.5	6.9 ± 2.2	<0.001
Serum Hb (g/dL)	10.8 ± 2.13	11.0 ± 2.0	10.5 ± 2.1	0.011	11.0 ± 2.0	9.9 ± 2.1	<0.001
Serum BUN (mg/dL)	31.3 ± 23.8	27.8 ± 19.1	34.5 ± 2.7	0.004	27.5 ± 18.8	45.1 ± 33.2	<0.001
Serum creatinine (mg/dL)	2.17 ± 4.53	2.35 ± 6.16	2.00 ± 2.13	0.420	1.75 ± 2.36	3.78 ± 8.77	0.031
Serum albumin (g/dL)	2.7 ± 0.7	2.9 ± 0.7	2.6 ± 0.7	<0.001	2.8 ± 0.7	2.5 ± 0.7	<0.001
UE shift, day/evening/night, *n* (%)	157 (35.3) / 163 (36.9) / 122 (27.6)	88 (41.9) / 75 (35.7) / 47 (22.4)	69 (29.7) / 88 (37.9) / 75 (32.3)	0.013	125 (35.9) / 132 (37.9) / 91 (26.1)	32 (34) / 31 (33) / 31 (33)	0.403
Agitation	155 (35.1)	81 (38.6)	74 (31.9)	0.142	127 (36.5)	28 (29.8)	0.227
Restrains	59 (13.3)	43 (20.5)	16 (6.9)	<0.001	47 (13.5)	12 (12.8)	0.852
Sedatives	288 (65.2)	143 (68.1)	145 (62.5)	0.218	228 (65.5)	60 (63.8)	0.761
Undergoing ventilator weaning at the time of UE	217 (49.1)	135 (65.5)	82 (35.7)	<0.001	192 (56.0)	25 (26.9)	<0.001
Intubation duration (hours) before UE	140.8 ± 169.4	111.1 ± 135.7	167.6 ± 191.4	<0.001	128.0 ± 138.2	188.1 ± 249.1	0.026

PaO₂, PaCO₂, and PaO₂/FiO₂ were derived from arterial blood gas analysis.

Expressed as mean ± SD (range), median (interquartile range), or *n* (%); APACHE II, Acute Physiology and Chronic Health Evaluation; TISS, Therapeutic Intervention Score System; COPD, chronic obstructive pulmonary disease; ESRD, end stage renal disease; BUN, blood urea nitrogen; UE, unplanned extubation.

### Outcomes of different UE groups

Overall, 52.5% (n = 232) experienced failed UE (reintubation within 48 h) (Table 2). Successful UE patients demonstrated shorter ICU stays (9.9 vs. 20.7 days, *p* < 0.001), shorter hospital stays (28.6 vs. 45.3 days, *p* < 0.001), reduced hospital costs (28.6 vs. 66.5 × 10^4^ new Taiwan dollars, p < 0.001), and lower mortality (9.1 vs. 33.2%, *p* < 0.001). Among survivor patients, shorter ICU stays and lower failed extubation rates were observed; however, hospital costs were higher.

**Table 2:** Outcomes of different UE groups.

Variables	Total patients (*n* = 442)	Successful (*n* = 210)	Failed (*n* = 232)	*p*-value	Survival (*n* = 348)	Non-survival (*n* = 94)	*p*-value
ICU stay (days)	15.6 ± 14.0	9.9 ± 9.3	20.7 ± 15.4	<0.001	14.0 ± 12.1	21.5 ± 18.3	<0.001
Hospital stay (days)	37.4 ± 30.1	28.6 ± 23.0	45.3 ± 33.4	<0.001	36.7 ± 28.4	40.1 ± 35.6	0.324
Hospital cost (NTD, x 10^4^)	51.4 ± 49.8	28.6 ± 23.0	66.5 ± 58.0	<0.001	45.0 ± 39.1	40.1 ± 73.0	<0.001
Failed UE	232 (52.5)				155 (44.5)	77 (81.9)	<0.001
Hospital mortality	94 (21.3)	17 (9.1)	77 (33.2)	<0.001			

Expressed as median (±) or *n* (%); UE, unplanned extubation; NTD, New Taiwan dollars.

### Risk factors for failed UE

Univariate analysis identified six variables significantly associated with failed UE, including the APACHE II score, respiratory rate (breaths/min), PEEP (cmH₂O), serum albumin (g/dL), total intubation time (hours) before UE, and undergoing ventilator weaning at the time of UE. Subsequent multivariate analysis identified five factors independently associated with failed UE: higher APACHE II scores, elevated respiratory rate and PEEP, longer total intubation duration, and not undergoing ventilator weaning at the time of UE (Table 3).

**Table 3:** Factors predicting reintubation within 48 h (failure) of UE using logistic regression model.

Variables	Crud OR (95%CI)	*p*-value	Adjusted OR (95%CI)	*p*-value
APACHE II score	1.051 (1.020–1.083)	0.001	1.054 (1.024–1.084)	<0.001
Respiratory rate (breaths/min)	1.051 (1.004–1.100)	0.032	1.055 (1.010–1.103)	0.017
PEEP (cmH2O)	1.190 (0.995–1.423)	0.056	1.211 (1.018–1.440)	0.031
Albumin (g/dL)	0.661 (0.451–0.967)	0.033	0.706 (0.489–1.020)	0.064
ET total time (hours) before extubation by self	1.003 (1.001–1.005)	0.003	1.003 (1.001–1.005)	0.001
Undergoing ventilator weaning at the time of UE	0.249 (0.143–0.249)	<0.001	0.260 (0.153–0.442)	<0.001

UE, unplanned extubation; APACHE II, Acute Physiology and Chronic Health Evaluation; TISS, Therapeutic Intervention Score System.

### Variables associated with mortality after UE

Univariate analyses identified 11 factors significantly associated with hospital mortality: APACHE II score, end-stage renal disease, liver cirrhosis, stroke, cancer, mean arterial pressure (mmHg), FiO₂ (%), minute ventilation (L/min), BUN (mg/dL), serum creatinine (mg/dL), ICU stay (days), and failed extubation. Subsequent multivariate analysis showed that hospital mortality was independently associated with eight factors: higher APACHE II scores, the presence of liver cirrhosis or cancer, higher FiO₂ or minute ventilation, elevated BUN levels, longer ICU stays, and failed extubation (Table 4). The primary causes of reintubation within 48 h were oxygenation failure (74.5%) and hemodynamic instability (9.1%) (Table 5).

**Table 4:** Mortality predictors of UE using logistic regression model.

Variables	Crud OR (95%CI)	*p*-value	Adjusted OR (95% CI)	*p*-value
APACHE II score	1.058 (1.008–1.111)	0.021	1.051 (1.008–1.095)	0.020
End stage renal disease	4.813 (1.008–19.670)	0.029	2.843 (0.968–8.347)	0.057
Liver cirrhosis	6.299 (1.646–24.108)	0.007	5.882 (1.737–19.918)	0.004
Stroke	3.232 (1.127–9.262)	0.029	1.534 (0.642–3.666)	0.336
Cancer	6.816 (2.417–19.221)	<0.001	4.949 (1.999–12.254)	0.001
Mean arterial pressure (mmHg)	0.959 (0.931–0.985)	0.002	0.984 (0.963–1.005)	0.139
FiO_2_ (%)	1.044 (1.011–1.078)	0.009	1.027 (1.003–1.052)	0.029
Minute ventilation (L/min)	1.299 (1.121–1.505)	0.001	1.159 (1.025–1.311)	0.019
BUN (mg/dL)	1.028 (1.005–1.050)	0.016	1.016 (1.001–1.031)	0.036
Serum creatinine (mg/dL)	1.269 (0.943–1.708)	0.116	1.203 (0.923–1.567)	0.172
ICU stay (days)	1.064 (1.032–1.097)	<0.001	1.034 (1.006–1.064)	0.018
Failed extubation	5.640 (3.202–9.935)	<0.001	2.406 (1.060–5.460)	0.036

UE, unplanned extubation; APACHE II, Acute Physiology and Chronic Health Evaluation; TISS, Therapeutic Intervention Score System; BUN, blood urea nitrogen; OR, odds ratio; CI, confidence interval.

**Table 5:** Causes for reintubation within 48 h (failure) of UE.

Causes	*n* = 232
Encephalopathy	10 (4.3%)
Excessive secretions	18 (7.8%)
Hemodynamic instability	21 (9.1%)
Oxygenation failure	173 (74.5%)
Upper airway obstruction	10 (4.3%)

## Discussion

To our knowledge, this study represents the largest and longest evaluation of a CQI program targeting UE in adult ICUs. Our previous study suggested that structured, multidisciplinary interventions could reduce UE incidence (12); the present 24-year analysis provides more extensive longitudinal evidence. After implementation of standardized procedures, revised sedation and weaning protocols, enhanced communication and restraint strategies, and a high-risk patient management task force, the UE rate declined from 6.82 to 0.65 episodes per 100 ventilated patients. The transient increase in 2005, coinciding with staff redistribution, underscores the sensitivity of UE events to system-level factors. The introduction of an artificial intelligence–based weaning prediction system in 2020 further supported extubation decision-making. Notably, despite the operational strain of the COVID-19 pandemic (25, 26), UE rates remained below 1 episode per 100 ventilated patients, demonstrating the durability of the continuous QI framework. However, because the interventions were implemented sequentially and overlapped over time, the relative contribution of each component cannot be disentangled. Therefore, these findings should be interpreted as reflecting the association between a sustained, bundled continuous QI framework and the observed decline in UE, rather than the effect of any single intervention. These findings are consistent with a recent analytical review by Anis et al. (27), which emphasized that UE risk factors are heterogeneous and support bundled, multidisciplinary preventive approaches rather than single interventions.

Recent studies have reported similar findings across different patient populations. Mekonnen et al. (20) reported that UE in pediatric ICUs was associated with adverse outcomes and modifiable care-related factors, supporting system-level prevention and high-risk patient surveillance. Multicenter pediatric and neonatal QI studies have also shown that standardized UE prevention bundles, structured workgroups, peer-to-peer learning, and targeted interventions can reduce UE events and severe harm (22, 28, 29). In contrast to these pediatric-focused studies, our study provides the longest and most comprehensive evaluation of a sustained quality improvement program for UE in adult ICUs, demonstrating that long-term, systemwide interventions can achieve substantial and durable reductions in UE.

Taken together, these findings place our results within a broader critical care QI framework. The sustained decline in UE suggests that prevention should be embedded into routine ICU systems rather than delivered as isolated interventions. Our continuous QI program reflected core QI principles, including standardization of high-risk processes, multidisciplinary teamwork, continuous event surveillance, root cause analysis, feedback to frontline staff, non-punitive accountability, and iterative refinement through PDCA cycles. Thus, UE prevention should be viewed as a dynamic safety process requiring ongoing monitoring, adaptation to system changes, and shared responsibility across ICU disciplines.

We further analyzed a 15-year cohort of 442 patients with UE and found that 52.5% required reintubation within 48 h, a rate consistent with previous reports ranging from 39.4 to 61.1% (14, 15, 30). Failed UE is clinically important because reintubation after UE has been associated with increased mortality, nosocomial pneumonia, prolonged ICU stay, and greater subsequent care needs (15, 31). Consistently, our study showed that patients with failed UE had longer ICU and hospital stays, higher hospital costs, and increased mortality. Similar adverse outcomes have also been reported in surgical ICU populations, including higher rates of tracheostomy, pneumonia, prolonged mechanical ventilation, and in-hospital mortality (32). These findings indicate that failed UE should be considered a high-risk event requiring early recognition, close post-event monitoring, and timely airway management.

We identified several factors associated with reintubation within 48 h of UE, including higher APACHE II scores, elevated respiratory rate and PEEP, longer intubation duration before UE, and not undergoing ventilator weaning at the time of UE. These factors reflect greater disease severity, limited respiratory reserve, and incomplete readiness for ventilator liberation. Because the transition from positive-pressure ventilation to spontaneous breathing may cause alveolar derecruitment, increased work of breathing, impaired gas exchange, and hemodynamic stress (33), sudden loss of the artificial airway in these patients may rapidly precipitate oxygenation failure, respiratory muscle fatigue, and acute respiratory failure. In addition, prolonged intubation duration before UE may reflect delayed readiness for extubation, accumulated respiratory burden, or missed opportunities for timely planned extubation, highlighting the need for closer multidisciplinary coordination. Our findings are consistent with previous studies that identified similar risk factors, including higher APACHE II scores, elevated PEEP, prolonged mechanical ventilation before extubation, and absence of weaning status (10, 15, 32, 34). In daily practice, these variables may serve as bedside warning signals after UE and should prompt immediate reassessment of respiratory reserve, oxygenation, work of breathing, and readiness for reintubation.

Variables associated with hospital mortality after UE in our study included higher APACHE II scores, liver cirrhosis or cancer, elevated FiO₂ or minute ventilation, higher BUN levels, prolonged ICU stay, and reintubation within 48 h, consistent with our previous reports (10, 11). These factors represent overlapping dimensions of risk, including greater illness severity, comorbidity burden, respiratory compromise, renal dysfunction or catabolic stress, prolonged critical illness, and acute post-UE deterioration. Liver cirrhosis and cancer may reduce physiologic reserve through pulmonary complications, systemic inflammation, immune dysfunction, frailty, organ dysfunction, and impaired respiratory recovery (35, 36). Elevated FiO₂ and minute ventilation reflect more severe oxygenation impairment and ventilatory demand, which may arise from ventilation-perfusion mismatch, intrapulmonary shunting, diffusion impairment, or hypoventilation (37). Higher BUN may indicate renal dysfunction, dehydration, protein catabolism, or multiorgan involvement, all of which are associated with poor outcomes in critically ill patients (38, 39). Finally, reintubation within 48 h may reflect early post-UE deterioration and identify patients vulnerable to periprocedural hypoxemia, aspiration, hemodynamic instability, ventilator-associated complications, and prolonged ICU treatment (15, 23, 40–42). Clinically, these findings support early post-UE risk stratification and proactive monitoring, particularly in patients with severe illness, high ventilatory support requirements, renal dysfunction, or major comorbidities.

This study has several limitations. First, it was conducted at a single, well-resourced tertiary medical center with structured multidisciplinary ICU coverage, a 2:1 patient-to-nursing staff ratio, and dedicated respiratory therapist support; therefore, differences in patient characteristics, staffing models, resources, workflows, and sedation or weaning practices may limit the generalizability and direct transferability of our findings. Second, because this was an observational study evaluating multiple sequential and overlapping continuous QI interventions over a long period, causality cannot be established, and the observed decline in UE incidence may have been influenced by secular improvements in ICU care, changes in patient case-mix, evolving sedation and ventilation practices, or other unmeasured system-level factors. The relative contribution of individual interventions could not be disentangled, and interrupted time-series or segmented trend analysis was not performed. Third, although UE events were prospectively registered, retrospective analysis of routinely collected clinical data may have resulted in missing or incomplete information, particularly because pre-event parameters may not have been uniformly recorded. Finally, we did not distinguish between self-extubation and accidental extubation because UE subtype was not systematically recorded. This distinction is clinically important because these entities may differ in mechanisms, risk profiles, and outcomes, and recent reports suggest that self-extubation may not increase mortality compared with planned extubation (34). Future studies should prospectively collect UE subtype data to clarify subtype-specific outcomes.

### Clinical implications

Our findings have practical implications for ICU management and quality improvement. ICU teams should adopt bundled, multidisciplinary strategies for UE prevention, including standardized endotracheal tube fixation, protocolized sedation and weaning assessment, restraint reassessment, structured handover communication, and high-risk patient surveillance. Routine risk stratification using APACHE II scores, ventilatory parameters, intubation duration, and weaning status may help identify patients at high risk for failed UE and guide closer post-event monitoring.

Hospitals may consider integrating UE prevention into continuous quality improvement systems rather than treating it as an isolated safety campaign. Practical approaches include daily extubation readiness assessment, respiratory therapist-led weaning surveillance, nursing checklists for tube security and agitation, early escalation pathways, non-punitive event reporting, root cause analysis, and feedback to frontline staff. Because ICU resources and staffing models vary across institutions, these strategies should be adapted to local capacity while preserving core QI principles, including standardized processes, multidisciplinary communication, risk surveillance, event review, and continuous feedback.

## Conclusion

A sustained continuous QI program was associated with a durable decline in UE incidence in adult ICUs over 24 years, suggesting the potential value of long-term, system-based quality improvement for preventable airway safety events. Among patients with UE, greater illness severity, higher ventilatory support requirements, renal dysfunction, prolonged ICU stay, major comorbidities, and reintubation were associated with increased mortality, emphasizing the importance of early risk stratification, proactive monitoring, and timely airway management after UE. Future multicenter studies are needed to validate these findings, assess the transferability of bundled UE prevention strategies across different ICU settings, and develop practical prediction tools for identifying patients at risk for failed UE or mortality after UE.

## Data Availability

The original contributions presented in the study are included in the article/Supplementary material, further inquiries can be directed to the corresponding authors.
