# Supplementary material for: Long-term impact of a quality improvement program on unplanned extubation and clinical outcomes in adult intensive care units: a 24-year single-center observational study
Source: Front Med (Lausanne). 2026 Jun 8;13:1772510. doi: 10.3389/fmed.2026.1772510 (PMC13284142; doi:10.3389/fmed.2026.1772510)
Supplement: Supplementary file 1 [file Supplementary_file_1.docx]

Supplementary Material


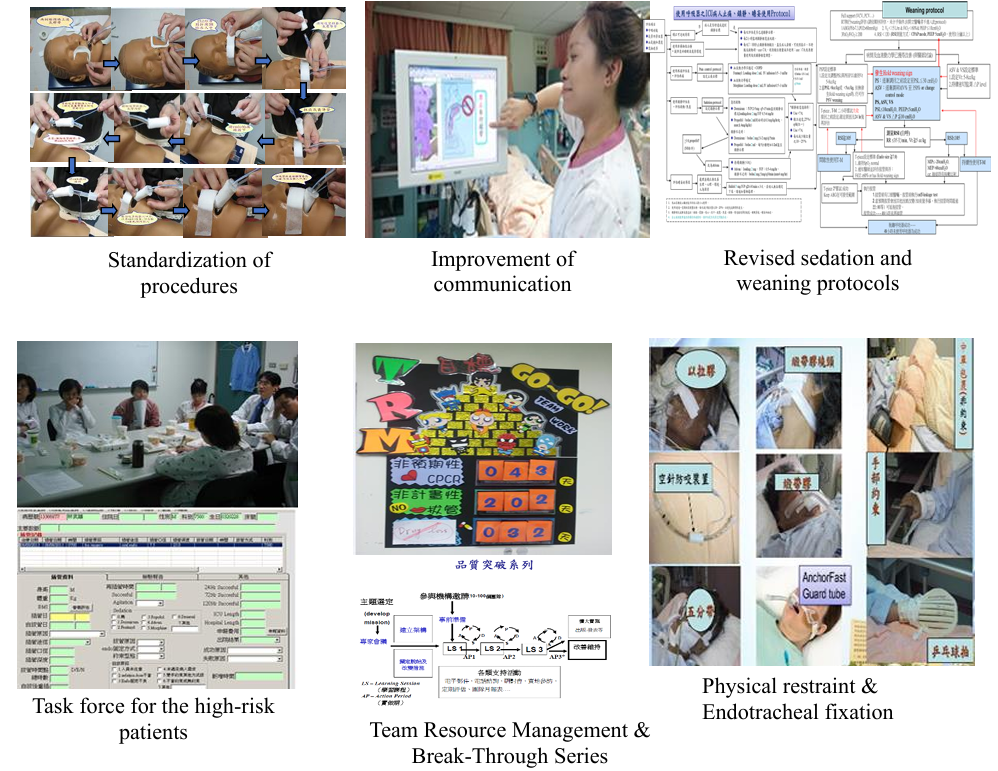


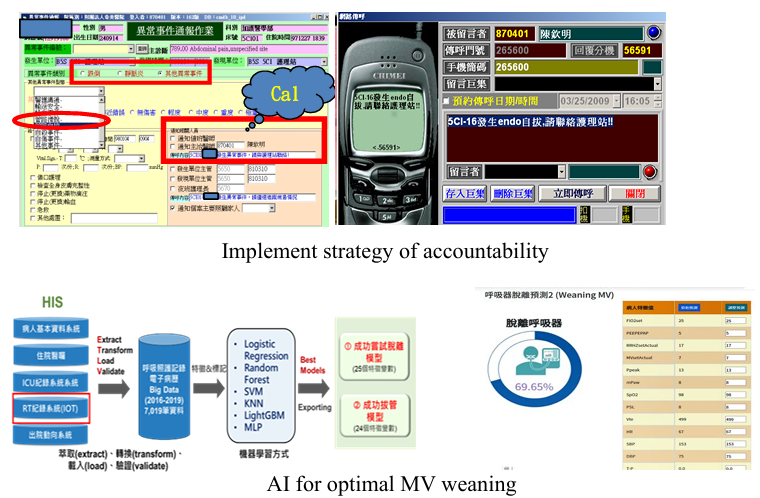


**Supplementary Figure 1.** continuous quality improvement (QI) concept

**Supplementary Table 1. Complete demographic, clinical, and most recent pre-extubation data of patients with unplanned extubation**

| **Variables** | **Total patients**  **(n=442)** | **Successful**  **(n=210)** | **Failed**  **(n=232)** | **p value** | **Survival**  **(n=348)** | **Non-survival**  **(n=94)** | ***p* value** |
| --- | --- | --- | --- | --- | --- | --- | --- |
| Age (years) | 65.4±15.4 | 65.1 ±1 5.3 | 65.7 ± 15.4 | 0.727 | 65.1 ± 16.0 | 66.7 ± 12.8 | 0.292 |
| Female patients | 141(31.9%) | 66 (31.4) | 75 (32.3) | 0.840 | 115 (33.0) | 26 (27.7) | 0.320 |
| Body mass index | 23.8±4.6 | 23.8 ± 4.6 | 23.9 ± 4.6 | 0.703 | 24.0 ± 4.6 | 23.3 ± 4.5 | 0.188 |
| APACHE II score | 18.2±8.9 | 15.3 ± 7.9 | 20.7 ± 9.0 | <0.001 | 16.7 ± 8..4 | 23.4 ± 8.7 | <0.001 |
| TISS Scales | 28.0±9.1 | 26.3 ± 8.2 | 29.4 ± 9.5 | <0.001 | 27.0 ± 8.7 | 31.3 ± 9.6 | <0.001 |
| Glasgow Coma Scales | 9.7±4 | 10 ± 4 | 9 ± 4 | <0.001 | 10 ± 4 | 10 ± 4 | 0.358 |
| Medical patients | 242(54.8) | 95 (45.2) | 147 (63.4) | <0.001 | 177 (50.9) | 65 (69.1) | 0.002 |
| Coronary artery disease | 108(24.4) | 57 (27.1) | 51 (22.0) | 0.207 | 84 (24.1) | 24 (22.2) | 0.780 |
| COPD | 57(12.9) | 25 (11.9) | 32 (13.8) | 0.554 | 45 (12.9) | 12 (12.8) | 0.966 |
| ESRD | 40(9.0) | 15 (7.1) | 25 (10.8) | 0.184 | 18(5.2) | 22(23.4) | <0.001 |
| Liver cirrhosis | 21(4.8) | 6 (2.9) | 15 (6.5) | 0.075 | 9 (2.6) | 12 (12.8) | <0.001 |
| Diabetes | 108(24.4) | 56 (26.7) | 52 (22.4) | 0.299 | 82 (23.6) | 26 (27.7) | 0.412 |
| Stroke | 126(28.5) | 63 (30.0) | 63 (27.2) | 0.508 | 108(31.0) | 18 (19.1) | 0.024 |
| Cancer | 50(11.3) | 20 (9.5) | 30 (12.9) | 0.259 | 25 (7.2) | 25 (26.6) | <0.001 |
| Mean arterial pressure (mmHg) | 96.5±17.9 | 97.4 ± 16.1 | 95.6 ± 19.6 | 0.352 | 98.1±16.6 | 89.7±21.8 | 0.004 |
| Heart rate (beats/min) | 90.8±18.1 | 89.2 ± 17.1 | 92.3 ± 19.0 | 0.072 | 90.3±17.2 | 92.9±21.2 | 0.277 |
| Respiratory rate (breaths/min) | 17.0±5.6 | 16.3 ± 5.5 | 17.6 ± 5.7 | 0.015 | 16.7 ± 5.6 | 18.0 ± 5.7 | 0.039 |
| pH | 7.425 ±0.070 | 7.433 ± 0.053 | 7.417 ± 0.081 | 0.014 | 7.434±0.052 | 7.392±0.107 | 0.001 |
| FiO_2_ (%) | 31 ±13 | 29 ± 7 | 34 ±17 | <0.001 | 30 ± 9 | 38 ± 21 | <0.001 |
| PaO_2_/FiO_2_ (mmHg) | 363.3 ±180.2 | 374.9±147.2 | 352.7 ± 205.2 | 0.206 | 374.3±173.7 | 321.7±198.4 | 0.014 |
| PaCO_2_ (mmHg) | 36.0 ±10.2 | 35.3 ± 7.1 | 36.7 ± 12.3 | 0.151 | 35.6±7.5 | 33.7 ±16.9 | 0.238 |
| Minute ventilation (L/min) | 8.5 ±3.1 | 8.0 ± 2.5 | 8.9 ± 3.5 | 0.002 | 8.2± 2.9 | 9.5 ± 3.4 | 0.001 |
| PEEP (cmH_2_O) | 5.9 ±1.8 | 5.5 ± 1.2 | 6.4 ± 2.0 | <0.001 | 5.7 ± 1.5 | 6.9 ± 2.2 | <0.001 |
| Serum Hb (g/dL) | 10.8 ±2.13 | 11.0 ± 2.0 | 10.5 ± 2.1 | 0.011 | 11.0 ± 2.0 | 9.9 ± 2.1 | <0.001 |
| Serum Hct (%) | 33.6 ±7.8 | 34.7 ± 7.9 | 32.7 ± 7.5 | 0.006 | 34.3 ± 7.2 | 31.1 ± 9.4 | 0.001 |
| Serum BUN (mg/dL) | 31.3 ±23.8 | 27.8 ± 19.1 | 34.5 ± 2.7 | 0.004 | 27.5 ± 18.8 | 45.1 ± 33.2 | <0.001 |
| Serum creatinine  (mg/dL) | 2.17 ±4.53 | 2.35 ± 6.16 | 2.00 ± 2.13 | 0.420 | 1.75 ± 2.36 | 3.78 ± 8.77 | 0.031 |
| Serum albumin (g/dL) | 2.7 ±0.7 | 2.9 ± 0.7 | 2.6 ± 0.7 | <0.001 | 2.8 ± 0.7 | 2.5 ± 0.7 | <0.001 |
| UE shift, day / evening / might, n (%) | 157 (35.3) / 163 (36.9) / 122 (27.6) | 88 (41.9) / 75 (35.7) / 47 (22.4) | 69 (29.7) / 88 (37.9) / 75 (32.3) | 0.013 | 125 (35.9) / 132 (37.9) / 91 (26.1) | 32 (34) / 31 (33) / 31 (33) | 0.403 |
| Agitation | 155(35.1) | 81 (38.6) | 74 (31.9) | 0.142 | 127(36.5) | 28(29.8) | 0.227 |
| Restrains | 59(13.3) | 43 (20.5) | 16 (6.9) | <0.001 | 47(13.5) | 12(12.8) | 0.852 |
| Sedatives | 288(65.2) | 143 (68.1) | 145 (62.5) | 0.218 | 228(65.5) | 60(63.8) | 0.761 |
| Use of Lorazepine | 30(6.8) | 10 (4.8) | 20 (8.6) | 0.107 | 17(4.9) | 13(13.8) | 0.002 |
| Undergoing ventilator weaning at the time of UE | 217(49.1) | 135 (65.5) | 82 (35.7) | <0.001 | 192 (56.0) | 25 (26.9) | <0.001 |
| Intubation duration (hours) before UE | 140.8 ±169.4 | 111.1±135.7 | 167.6±191.4 | <0.001 | 128.0 ± 138.2 | 188.1 ± 249.1 | 0.026 |

PaO₂, PaCO₂, and PaO₂/FiO₂ were derived from arterial blood gas analysis.

Expressed as mean ± SD (range), median (interquartile range) or n (%); APACHE II = Acute Physiology and Chronic Health Evaluation; TISS = Therapeutic Intervention Score System; COPD = chronic obstructive pulmonary disease; ESRD = end stage renal disease; BUN = Blood Urea Nitrogen; UE = unplanned extubation
